# Supplementary material for: Deletion of wfs1 Impairs Oligodendrocyte Precursor Cells Dorsal Distribution and Myelination Through the wfs1-hmgcs1 Axis in Zebrafish
Source: Biology (Basel). 2026 Aug 16;15(16):1402. doi: 10.3390/biology15161402 (PMC13510151; doi:10.3390/biology15161402)
Supplement: Supplementary file 1 [file biology-15-01402-s001.zip › File S1-Transcriptome sequencing data and differentially expressed genes (DEGs) in wfs1-- zebrafish at 5 dpf/2.KEGG/wfs1KOvsWT/ALL/src/dre00230.html]

dre00230


- dre:100330864

- Up regulated genes

100330864(6.66489299727258)

- dre:560827

- Up regulated genes

560827\_1(4.8362689625617)

- dre:334431

- Up regulated genes

334431\_1(1.27207238534102)

- dre:568726

- Up regulated genes

568726(1.14419230813088)
- dre:560807

- Up regulated genes

560807(1.71247301071773)

- dre:447905

- Up regulated genes

447905(1.08491752479688)

- dre:100330864

- Up regulated genes

100330864(6.66489299727258)

- dre:334431

- Up regulated genes

334431\_1(1.27207238534102)

- dre:333997

- Up regulated genes

333997(1.82688585400469) 333997\_1(1.97878985903326)

- dre:402953

- Up regulated genes

402953\_1(1.71924648864672)

- dre:402953

- Up regulated genes

402953\_1(1.71924648864672)

- dre:553639

- Up regulated genes

553639\_1(3.56124711998536)

- dre:550560

- Up regulated genes

550560\_1(1.24185328918673)

- dre:402953

- Up regulated genes

402953\_1(1.71924648864672)

- dre:402953

- Up regulated genes

402953\_1(1.71924648864672)

- dre:402953

- Up regulated genes

402953\_1(1.71924648864672)

- dre:140425

- Up regulated genes

140425(1.92392776077717)

- dre:777719

- Down regulated genes

777719(-4.40662443486041)

- dre:777719

- Down regulated genes

777719(-4.40662443486041)

- dre:100005067

- Down regulated genes

100005067\_1(-1.86646622375553)

- dre:402953

- Up regulated genes

402953\_1(1.71924648864672)

- dre:568726

- Up regulated genes

568726(1.14419230813088)
- dre:560807

- Up regulated genes

560807(1.71247301071773)

- dre:550560

- Up regulated genes

550560\_1(1.24185328918673)

- dre:334431

- Up regulated genes

334431\_1(1.27207238534102)

- dre:402953

- Up regulated genes

402953\_1(1.71924648864672)

Close
